# Physical Activity Indicators Among Children and Adolescents in Lebanon, Qatar, and the United Arab Emirates: Comparative Synthesis of Active Healthy Kids Report Card Data From 1998 to 2022

**DOI:** 10.2196/85998

**Published:** 2026-03-24

**Authors:** Ameneh Baghestani, Lina Majed, Merilyn Lock, Ali Alrahma, Patrick Abi Nader, Suzan Sayegh, Abdulla Saeed Al-Mohannadi, Javaid Nauman, Salomé Aubert, Mark S Tremblay, Tom Loney

**Affiliations:** 1 College of Medicine, Mohammed Bin Rashid University of Medicine and Health Sciences, Dubai Health, Dubai, United Arab Emirates, 971 43838737; 2 Department of Sports and Computer Science, Universidad Pablo de Olavide, Seville, Andalusia, Spain; 3 College of Health and Life Sciences, Hamad Bin Khalifa University, Doha, Qatar; 4 School of Health and Environmental Studies, Hamdan Bin Mohammed Smart University, Dubai, United Arab Emirates; 5 Institute of Public Health, College of Medicine and Health Sciences, United Arab Emirates University, Al Ain, United Arab Emirates; 6 Faculté des sciences de l'activité physique, Université de Sherbrooke, Sherbrooke, QC, Canada; 7 Modern University for Business and Science, Beirut, Lebanon; 8 Shift Wellness Institute, Vancouver, WA, United States; 9 World Innovation Summit for Health (WISH), Qatar Foundation, Doha, Qatar; 10 Department of Circulation and Medical Imaging, Faculty of Medicine and Health Sciences, Norwegian University of Science and Technology, Trondheim, Trøndelag, Norway; 11 Active Healthy Kids Global Alliance, Ottawa, ON, Canada; 12 Children's Hospital of Eastern Ontario Research Institute, Ottawa, ON, Canada

**Keywords:** physical activity surveillance, physical education, sedentary lifestyle, sports, children and adolescents, Middle East

## Abstract

**Background:**

Physical inactivity and sedentary behavior are important modifiable risk factors for noncommunicable diseases. High prevalences of physical inactivity among children and adolescents continue to represent a significant public health challenge globally, with approximately two-thirds of children worldwide not achieving the recommended daily amount of physical activity (PA). Countries in the Middle East exhibit some of the highest levels of physical inactivity and sedentary behavior, which contribute to the increasing rates of obesity among children and adolescents.

**Objective:**

This study aims to provide a comparative synthesis of PA indicators among children and adolescents in Lebanon, Qatar, and the United Arab Emirates (UAE) based on the Active Healthy Kids Global Alliance (AHKGA) PA Report Cards and further compare the findings with regional and global trends.

**Methods:**

Data were synthesized from previous AHKGA PA Report Cards published by researchers from Lebanon, Qatar, the UAE in the years 2016 (Global Matrix 2.0; 1998‐2014 data), 2018 (Global Matrix 3.0; 2016‐2017 data) and 2022 (Global Matrix 4.0; 2017‐2022 data). We evaluated 10 key PA indicators across these countries to identify trends and gaps in PA levels among children and adolescents. These findings were further compared with regional and global data gathered and published in previous iterations of the AHKGA Global Matrix.

**Results:**

Based on data collected between 1998 and 2022, less than one-third (15%‐33%) of children and adolescents in Lebanon, Qatar, and the UAE achieved the recommended daily average of 60 minutes of moderate- to vigorous-intensity PA. Additionally, more than one-half (45%‐74%) of children and adolescents exceeded the recommended limit of 2 hours of recreational screen time per day. Overall, boys were more physically active than girls; however, PA levels declined with increasing age. Other behavioral indicators such as participation in organized sports and active transportation revealed insufficient PA levels. The results were slightly better for sources of influence indicators especially with the opportunities provided by schools and governments. Compared with global estimates, PA levels in the Middle Eastern countries were similar to the averages observed across Asian countries participating in the AHKGA; however, they were generally lower than PA levels in other regions of the world.

**Conclusions:**

Data from a 25-year period show consistently low levels of PA and high levels of sedentary behavior among children and adolescents from these 3 Arab Middle Eastern countries. Despite governmental investments in implementation of PA initiatives, there seems to be a lag in eliciting increases in PA at the population level. Evidence points to a critical need for behavioral and lifestyle modifications among children and adolescents. These concerns are exacerbated by a lack of national surveillance systems and evidence-based policy interventions to improve PA levels.

## Introduction

### Background and Rationale

Physical activity (PA) is an important factor for preventing and managing noncommunicable diseases, as well as enhancing mental health, quality of life, and overall well-being [1]. The high prevalence of physical inactivity among children and adolescents continues to represent a global health challenge, with approximately two-thirds of children worldwide failing to achieve the minimum recommended level of daily PA (ie, at least an average of 60 minutes of moderate- to vigorous-intensity PA [MVPA]) [2]. Moreover, countries in the Middle East report some of the highest levels of physical inactivity and sedentary behavior [3,4]. This trend is further reflected in the increasing rates of obesity among children, adolescents, and adults in the region [5]. Given that physical inactivity is an independent risk factor for various chronic diseases, PA surveillance and intervention should be a top priority for public health agencies, policymakers, and governments [6,7].

In 2005, a team of Canadian researchers developed a Report Card that provided a comprehensive assessment of PA among Canadian children and adolescents, with the aim of identifying areas of concern and advocating for action to improve their PA levels [8,9]. The Report Card included indicators related to PA in different domains, and each indicator was assigned a letter grade (A to F) based on the best available evidence, with “A” representing the highest level of achievement and “F” representing the lowest. For 20 years, Active Healthy Kids Canada and ParticipACTION have worked to inspire the country to engage all children and adolescents in PA, and the Canadian Report Card was released annually between 2005 and 2016 and periodically thereafter.

Building on the success of the Canadian model, the Active Healthy Kids Report Card initiative expanded globally to an international project encompassing 15 countries producing PA Report Cards as part of the Global Matrix 1.0 in 2014 [10]. Since then, the project has continued to grow and include more countries from all regions (Global Matrix 2.0, 2016, 38 countries [11]; Global Matrix 3.0, 2018, 49 countries [12]; Global Matrix 4.0, 2022, 57 countries [13]). The Active Healthy Kids Global Alliance (AHKGA) leads the Global Matrix initiative and has made significant efforts to gather evidence-based data on PA indicators among children and adolescents worldwide [14,15]. As a result, the individual country Report Cards have formed a source of consolidated research knowledge on PA, with contributions to academia, research, education, health, policy, and related fields [16,17]. The primary goal of the AHKGA “Global Matrix” initiative is to raise awareness, develop capacity, and exchange knowledge and strategies, as well as expand PA surveillance of children and adolescents aged 5 years through 17 years across different jurisdictions, countries, and continents. The initiative follows a systematic assessment process; it involves reviewing key indicators and influencing factors using a predefined methodology and grading rubric. This process, steered by the AHKGA, is conducted periodically (every 2 years to 4 years). The AHKGA Global Matrix is currently the only worldwide initiative that comprehensively assesses PA among children and adolescents, covering a diverse range of indicators. This study presents the first comparative synthesis across participating Arab Middle Eastern AHKGA countries.

Data gathered from various participating countries over the past decade (ie, Global Matrix 2.0, 3.0, and 4.0) have consistently indicated low levels of PA across most domains [18]. Without significant changes in behaviors and lifestyles, current and future generations may face increased risks of noncommunicable diseases as they transition into adulthood [3]. In Asia, the number of countries taking part in the AHKGA initiative has increased over the years (9 in Global Matrix 2.0, 12 in Global Matrix 3.0, and 16 in Global Matrix 4.0), demonstrating the region’s enthusiasm for the promotion of PA. However, the overall PA levels have been relatively low throughout the years in most Asian countries [19,20]. As such, gaining additional insights on the region’s PA landscape is important for shaping targeted interventions and informing policy decisions to enhance PA levels and outcomes among children and adolescents. Cross-country analysis of data gathered by the AHKGA can help identify trends and influencing factors specific to the region, as well as reveal gaps in existing surveillance systems [21]. Within the Middle East region, participation in the AHKGA initiative has so far been limited to a few countries, with Lebanon, Qatar, and the United Arab Emirates (UAE) being the only Arab nations that have independently developed Report Cards.

### Objectives

This study aimed to synthesize and compare evidence from the PA Report Cards developed by these Arab Middle Eastern countries. Our study specifically focused on Arab nations, with the objective of evaluating trends and identifying gaps in PA indicators among children and adolescents in countries with similar cultural backgrounds but different socioeconomic and political climates. It also examined the relationships between socioeconomic and geographic factors and the levels of PA, thereby enabling a comparison of findings with those of other regions and global averages.

## Methods

### Study Design

We used a harmonized comparative approach to evaluate PA indicators among children and adolescents across 3 Arab countries in the Middle East region (Lebanon, Qatar, UAE) using previous PA Report Card outcomes published as part of the AHKGA Global Matrix initiative. We further compared these results with regional and global findings.

### Variables

We evaluated 10 key indicators of PA related to children’s and adolescents’ behaviors, fitness, and sources of influence in this study. The indicators, as defined by the AHKGA, included (1) Overall Physical Activity, (2) Organized Sport and Physical Activity, (3) Active Play, (4) Active Transportation, (5) Physical Fitness (added in Global Matrix 3.0 onward), (6) Sedentary Behavior, (7) Family and Peers, (8) School, (9) Community and Environment, and (10) Government.

### Data Sources

The AHKGA Report Card archives were checked for all Arab Middle Eastern countries that were involved in any Global Matrix initiative (1.0 to 4.0). Relevant publications outlining the results of each of the published Report Cards were identified by collaborating authors who were involved in the original projects. This study included data from the Report Cards published in Global Matrix 2.0 2016 (data from 1998‐2014) [22,23], Global Matrix 3.0 2018 (data from 2016‐2017) [24-26], and Global Matrix 4.0 2022 (data from 2017‐2022) [27,28] by Lebanon, Qatar, and the UAE. Participation among these countries varied across the years; the UAE participated in 3 iterations of the Global Matrix (2.0 to 4.0), Qatar participated in Global Matrix 2.0 and 3.0, and Lebanon participated in Global Matrix 3.0 and 4.0. A total of 7 PA Report Cards were identified, and the results were evaluated in our study.

During the development of the Report Cards for each respective iteration (2016, 2018, 2022), teams from each country systematically gathered the most valid and reliable evidence and relevant data from the given time periods (ie, Global Matrix 2.0 data from pre-2016, Global Matrix 3.0 data from 2016‐2017, and Global Matrix 4.0 data from 2017‐2022). The countries’ Report Card teams included researchers, clinicians, and policymakers from universities, educational authorities, public health departments, and government health authorities from each country. Data sources included local, national, and international studies; national surveys; and official reports. Data were then synthesized by Report Card teams, and a consensus was established regarding the grading of each indicator. The grading was based on specific benchmarks, with the assigned grades corresponding to the proportion of children who achieved the benchmark (Table 1). Grading in the 2016 Report Cards was as follows: A=81% to 100%; B=61% to 80%; C=41% to 60%; D=21% to 40%; F=0% to 20%; INC =incomplete data. However, from 2018 onward, each grade band was further subdivided (Table 2). Report Card grades for each country, including rationale and references, were audited and reviewed by the AHKGA prior to final approval and release.

**Table 1.** Indicators and benchmarks used to guide the Report Card grading for Global Matrix 2.0 in 2016 (data from 1998‐2014), Global Matrix 3.0 in 2018 (data from 2016‐2017), and Global Matrix 4.0 in 2022 (data from 2017‐2022).

Indicator	Benchmark
Overall Physical Activity	% of children and adolescents who meet the Global Recommendations on Physical Activity for Health, which recommend that children and adolescents accumulate at least 60 minutes of moderate- to vigorous-intensity PA^a^ per day on average % of children and adolescents meeting the guidelines on at least 4 days a week (when an average cannot be estimated)
Organized Sport and Physical Activity	% of children and adolescents who participate in organized sport or PA programs
Active Play	% of children and adolescents who engage in unstructured or unorganized active play at any intensity for more than 2 hours a day % of children and adolescents who report being outdoors for more than 2 hours a day
Active Transportation	% of children and adolescents who use active transportation to get to and from places (eg, school, park, mall, friend’s house)
Sedentary Behavior	% of children and adolescents who meet the Canadian Sedentary Behavior Guidelines for 5- to 17-year-olds, which is no more than 2 hours of recreational screen time per day
Physical Fitness	Average percentile achieved on certain physical fitness indicators based on the normative values published by Tomkinson et al [29]
Family and Peers	% of family members (eg, parents, guardians) who facilitate PA and sport opportunities for their children (eg, volunteering, coaching, driving, paying for membership fees and equipment) % of parents who meet the Global Recommendations on PA for Health, which recommend that adults accumulate at least 150 minutes of moderate-intensity aerobic PA throughout the week or do at least 75 minutes of vigorous-intensity aerobic PA throughout the week or an equivalent combination of moderate- and vigorous-intensity PA % of family members (eg, parents, guardians) who are physically active with their kids % of children and adolescents with friends and peers who encourage and support them to be physically active % of children and adolescents who encourage and support their friends and peers to be physically active
School	% of schools with active school policies (eg, daily PE^b^, daily PA, recess, “everyone plays” approach, bike racks at school, traffic calming on school property, outdoor time) % of schools where the majority (≥80%) of students are taught by a PE specialist % of schools where the majority (≥80%) of students are offered the mandated amount of PE (for the given state, territory, region, or country) % of schools that offer PA opportunities (excluding PE) to the majority (>80%) of their students % of parents who report their children and adolescents have access to PA opportunities at school in addition to PE classes % of schools with students who have regular access to facilities and equipment that support PA (eg, gymnasium, outdoor playgrounds, sporting fields, multipurpose space for PA, equipment in good condition)
Community and Environment	% of children or parents who perceive their community or municipality is doing a good job at promoting PA (eg, variety, location, cost, quality). % of communities or municipalities that report they have policies promoting PA % of communities or municipalities that report they have infrastructure (eg, sidewalks, trails, paths, bike lanes) specifically geared toward promoting PA % of children or parents who report having facilities, programs, parks, and playgrounds available to them in their community % of children or parents who report living in a safe neighborhood where they can be physically active % of children or parents who report having well-maintained facilities, parks, and playgrounds in their community that are safe to use
Government	Evidence of leadership and commitment in providing PA opportunities for all children and adolescents Allocated funds and resources for the implementation of PA promotion strategies and initiatives for all children and adolescents Demonstrated progress through the key stages of public policy making (ie, policy agenda, policy formation, policy implementation, policy evaluation, and decisions about the future)

a PA: physical activity.

b PE: physical education.

**Table 2.** Report Card grading rubric used in the Global Matrix 2.0^a^ in 2016 (data from 1998‐2014), Global Matrix 3.0 in 2018 (data from 2016‐2017), and Global Matrix 4.0 in 2022 (data from 2017‐2022).

Grade	Interpretation
A+	94%‐100%
A	87%‐93% (We are succeeding with a large majority of children and adolescents.)
A–	80%‐86%
B+	74%‐79%
B	67%‐73% (We are succeeding with well over half of children and adolescents.)
B–	60%‐66%
C+	54%‐59%
C	47%‐53% (We are succeeding with about half of children and adolescents.)
C–	40%‐46%
D+	34%‐39%
D	27%‐33% (We are succeeding with less than half but some children and adolescents.)
D–	20%‐26%
F	<20% (We are succeeding with very few children and adolescents.)
INC^b^	Incomplete—insufficient or inadequate information to assign a grade

a Grading in the 2016 Report Cards was as follows: A=81%-100%; B=61%-80%; C=41%-60%; D=21%-40%; F=0%-20%.

b INC: incomplete data.

In this study, we incorporated references to the published results of the Report Cards from each country over the years [22-28]. We also relied on the interpretations made by the Report Card teams to generate a comprehensive table with the assigned grades and percentages based on the previous publications. In certain instances, we cited additional sources that did not play a direct role in determining the indicator’s grade at the time that the Report Card was issued (eg, governmental policies, future plans, and suggested strategies for improvement); however, they remain relevant to the context of the grading. This approach helped us provide thorough interpretations while ensuring that the grading process was based on information from the published Report Cards. Additionally, the most recent Report Cards (2022) included the top 3 priorities for improving 10 key PA indicators, as identified by Report Card teams in each country. Building on these priorities, we provided key applicable recommendations for the indicators to support the improvement of PA within the 3 Arab Middle Eastern countries.

Sociodemographic characteristics of the countries were obtained from the latest (2023 and 2024) Human Development Reports issued by the United Nations and the World Bank [30,31], including the Human Development Index (HDI), gross domestic product (GDP) per capita, Gender Inequality Index, Gini Index, expenditure on health, life expectancy at birth, mean years of schooling, total population, population density, urban population, and average mean temperature [32]. They were used to assess and interpret grade variations between these countries and in relation to other regions of the world.

### Ethical Considerations

This study used secondary data from published papers and did not require institutional review board review as the data were publicly available. Specifically, data were collected from Global Matrix and Report Card papers published in the Journal of Physical Activity and Health [11-13] as well as the AHKGA website [14] for Global Matrix 2.0, 3.0, and 4.0. This article presents a comparative secondary data analysis of the aforementioned publications for the 3 participating countries. As such, no ethical approval was required for conducting the study. The referenced publications included data from local, national, and international studies; national surveys; and official reports. References to these data sources can be found in the relevant original Report Card publications as cited in the Methods section [22-28]. Further information about the process of Report Card development in each participating country can be found in the original Global Report Card publications [11-13].

## Results

### Overview

To contextualize the findings across these Arab Middle Eastern countries, sociodemographic and economic characteristics were first examined (Table 3). Qatar (0.886) and the UAE (0.940) had very high (≥0.800) HDIs [30,33]; Lebanon (0.752) had a high (0.700‐0.799) HDI [30,34]. As of the year 2024, the UAE had the largest population among the 3 countries, with approximately 11 million residents, and the lowest population density (142 people/km^2^ of land area) and urban population percentage (88%). Public health expenditure as a percentage of GDP was the highest in Lebanon (5.74%) and the lowest in Qatar (2.18%). Life expectancy at birth ranged from 77.8 years in Lebanon to 82.9 years in the UAE. The lowest Gini Index (least income inequality) was observed in the UAE (26.4), and the highest (greatest income inequality) was in Qatar (35.1) [31].

**Table 3.** Sociodemographic characteristics of the 3 participating Arab Middle Eastern countries from the most recent available data.

Sociodemographic variable	Country		
Lebanon	Qatar	UAE^a^
Human Development Index	0.752	0.886	0.940
GDP^b^ per capita (US $)	3477.70	127,711.10	49,377.60
Gender Inequality Index	0.036	0.195	0.040
Gini Index	31.8	35.1	26.4
Expenditure on health (% of GDP)	5.74	2.18	4.68
Life expectancy at birth (years), mean	77.8	82.4	82.9
Schooling duration (years), mean	10.4	10.8	13.0
Population (millions), n	5.8	2.9	10.9
Population density (people/km^2^)	562	231	142
Urban population, %	90	99	88
Temperature (°C), mean	16.0	28.3	28.9

a UAE: United Arab Emirates.

b GDP: gross domestic product.

In total, 37 letter grades (37/66, 56%) and 29 incomplete (INC) grades (29/66, 44%) were assigned across all 7 Report Cards (Table 4). The indicator with the highest grade was Government (average of B+), and the indicator with the lowest grade was Overall Physical Activity (average of D/F). Active Play, Physical Fitness, and Community and Environment were marked INC throughout all Report Cards. The grades are explained in the following sections, except for indicators with INC grades, which are not included in the rest of the Results section.

**Table 4.** Grades assigned to the 10 common physical activity (PA) indicators for Global Matrix 2.0 in 2016 (data from 1998-2014), Global Matrix 3.0 in 2018 (data from 2016-2017), and Global Matrix 4.0 in 2022 (data from 2017-2022) for Lebanon, Qatar, and the United Arab Emirates (UAE).

Common PA indicators	GM^a^ 2.0 (2016)^b^, grade (%)		GM 3.0 (2018), grade (%)			GM 4.0 (2022), grade (%)	
	UAE (1998-2014)	Qatar (2004-2014)	UAE(2016)	Qatar(2016)	Lebanon (2016-2017)	UAE (2017-2021)	Lebanon (2018-2022)
Overall Physical Activity	F–/D– (17‐28)	F (15)	F (16)	D (32)	D (33)	F (19)	D– (21)
Organized Sport and Physical Activity	INC^c^	D (30)	INC	D+ (48)^d^	F (5)	INC	INC
Active Play	INC	INC	INC	INC	INC	INC	INC
Active Transportation	F–/D– (19‐21)	N/A	INC	N/A	D (26‐37)^d^	F (5)	D+ (37)
Sedentary Behavior	C– (49‐62)^d^	D (<30)	C– (40)	D+ (35‐45)^d^	C– (46)	D– (26)	C (55)^d^
Physical Fitness	—^e^	—	INC	INC	INC	INC	INC
School	D (28)	INC	D– (26)	C (61‐67)^d^	D (22‐58)^d^	A– (100)^d^	D (32)
Family and Peers	C– (62)^d^	D	INC	INC	INC	D– (20‐26)	INC
Community and Environment	INC	INC	INC	INC	INC	INC	INC
Government	B+	B	B+	B+	C+	B+	D

a GM: Global Matrix.

b Minus sign in the 2016 Report Card grading scheme indicates variations in subgroups based on age, gender, nationality, socioeconomic status, and geographic location.

c INC: incomplete.

d Percentages may not correspond to final grades, as final grades were determined by expert consensus.

e Not applicable: The Physical Fitness indicator was added in Global Matrix 3.0 onward.

Additionally, during each iteration of the Global Matrix (2016, 2018, 2022), participating countries had the choice to include supplementary indicators in their respective Report Cards, as deemed beneficial to enhance the value of their results. Due to this, some variations in the indicators were observed across the different countries and Report Cards. For example, Qatar’s Report Card team decided not to evaluate Active Transportation and considered it “Not Applicable” due to road safety issues and the prevalence of high temperatures during much of the year. Instead, they included Obesity and Dietary Habits as additional indicators in their 2016 Report Card. Likewise, Lebanon included Weight Status in addition to the common indicators in their 2018 Report Card and Sleep and Body Mass Index in their 2022 Report Card.

### Overall Physical Activity

Across all 3 countries, the proportions of children and adolescents meeting the recommended PA levels (ie, minimum of 60 minutes of MVPA per day on average) varied from 15% to 33%, with the average grades ranging from F to D. In the UAE, data from 1998 to 2021 showed that most children and adolescents (72%‐84%) were physically inactive. The first UAE Report Card (2016), which was based on the largest amount of data collected (1998‐2014), revealed 17% to 28% of children and adolescents achieved the recommended daily PA, with a noticeable difference between boys (23%‐24%) and girls (13%‐16%). The World Health Organization (WHO) Global School-based Health Survey (GSHS) [35] and other studies with smaller sample sizes were used to inform this indicator, resulting in a split grade of D−/F−, with the minus sign indicating variations in subgroups. Overall Physical Activity remained low in subsequent UAE Report Cards, with only 16% of children and adolescents (21% of boys; 11% of girls) in 2018 and 19% of children and adolescents (27% of boys; 8% of girls) in 2022 accumulating sufficient PA. Likewise, in Qatar, only 15% of children and adolescents (30% of boys; 10% of girls) met the recommended daily PA as reported in their first and most comprehensive Report Card issued in 2016. There was a notable increase in the following Qatar Report Card (2018), with PA levels ranging between 25% and 39% among children and adolescents. The final average grade assigned to Overall Physical Activity in the 2018 Report Card by Qatar was D (32%). The Lebanese Report Cards for 2018 and 2022 reported comparable results, with average grades of D (33%) and D− (21%) assigned in those respective years. Like Qatar and the UAE, their evaluation primarily relied on the WHO GSHS data using self-report PA estimates. Boys had higher levels of PA (29%) than girls (14%), and students attending private schools (23%) appeared to be more active than students in public schools (17%) in Lebanon. In all 3 countries, the Overall Physical Activity estimates were mostly derived from self-reported questionnaires, while device-based (accelerometer) assessments were limited.

### Organized Sport and Physical Activity

In Lebanon, the proportion of children and adolescents participating in events or competitions administered by the Ministry of Youth and Sports was extremely low, leading to an F grade (5%) in 2018. This may be due to a lack of public sport infrastructure and investment in community-level sport programs. Their subsequent Report Card (2022) graded this indicator INC. In Qatar and the UAE, governmental support and investment in infrastructure were and continue to be higher than in Lebanon. In 2016, Qatar’s GSHS national survey found that approximately 30% of children and adolescents regularly participated in at least one organized sport annually. Qatar also held a National Sports Day and had several initiatives aimed at promoting sport participation, which contributed to a grade D in this area. This grade improved to D+ in 2018, when nearly 48% of children and adolescents participated in organized sports. Additionally, 58% of boys and 42% of girls in Qatar were registered with sport clubs. The UAE marked this indicator as INC throughout all Report Cards.

### Active Transportation

Of the 3 included countries, Lebanon reported the highest active transportation use (26%‐37%; grade D). The proportion remained similar (37%), but the grade increased from D (2018) to D+ in 2022. Additionally, sex-based differences were observed (42% of boys and 32% of girls) in the 2017 GSHS national survey results. The UAE’s evaluation of Active Transportation in 2016 was derived from 2005 and 2010 GSHS national reports, when data showed that 19% to 21% of adolescents walked or cycled to school at least once during the week (grade F−/D−). The minus sign added to the grade in the 2016 Report Card was due to sex-based differences (29% of boys and 16% of girls in 2005; 29% of boys and 15% of girls in 2010). No data were available for the following (2018) UAE Report Card; however, the 2022 UAE Report Card used data from an online survey run by the Knowledge and Human Development Authority in Dubai. The survey of 33,784 parents indicated that only 5% of children and adolescents in Dubai used active transportation modes to travel to and from school (4.8% walked; 0.2% cycled) [27]. Based on these findings, the indicator received an F in 2022. Qatar did not include Active Transportation in their Report Cards, as they considered it to be not applicable for assessment in their country due to road safety issues and the predominantly hot climate.

### Sedentary Behavior

All 3 countries have faced ongoing challenges in reducing sedentary behavior. The UAE Report Card showed 62% (2005) and 49% (2010) of children achieving the recommended Canadian Sedentary Behavior Guidelines of screen time exposure (ie, no more than 2 hours of recreational screen time per day), with variations according to age and sex. The indicator was assigned a C− in the UAE in the first two Report Cards (2016 and 2018), with the minus sign indicating variations in subgroups in 2016. The numbers declined over time to 40% in 2018 (C–) and only 26% in 2022 (D−). In Qatar, the 2016 Report Card showed that less than one-third of children and adolescents met the recommended daily recreational screen time, which corresponded to a D grade. These figures improved slightly to 35% for children and 45% for adolescents (D+) in 2018. Qatar did not participate in the 2022 Report Card; therefore, updated data were unavailable. The 2018 Lebanese Report Card showed that approximately 40% of children and 52% of adolescents met the screen time recommendations, resulting in an average grade of C− (46%). Similar findings were reported in 2022, with 55% of adolescents meeting the recommendations.

### School

In the UAE, due to limited access to national data on school distribution, the benchmark used for this indicator varied between (1) the proportion of children and adolescents attending ≥3 physical education (PE) classes per week (approximately 150 minutes) and (2) the proportion of schools where the majority (≥80%) of students were taught by PE specialists. In 2016 and 2018, the UAE reported that slightly more than one-quarter (26%‐28%) of adolescents adhered to the mandated PE requirements and that participation decreased as age increased. The 2022 Report Card reflected updated figures, with an A− grade; however, this grade was based solely on data confirming that all children and adolescents in Dubai received PE classes taught by specialists. The minus sign in the 2022 Report Card was added to acknowledge the difference in the frequency of weekly PE classes across various schools. Although several components of the UAE schools (such as all PE teachers holding bachelor’s degrees in PE and the adequate availability of PA facilities) would typically warrant a high grade for this indicator, only 79% of children reported receiving more than 100 minutes of PE classes per week. In Qatar, School was marked INC in 2016 due to insufficient data. In 2018, it was graded C, indicating that 67% of children participated in PE classes and 61% of schools offered support for PA programs or initiatives such as “Qatar Active Schools” and “Health Promoting Schools.” It is worth noting here that, at the time, many PE teachers in Qatar were not classified as specialists. Therefore, schools in these circumstances would not have been counted, even if their PE classes were delivered adequately. In Lebanon, School was graded D in 2018 (22%) and 2022 (32%). In both instances, the results were based on self-reported participation of school students in the number of weekly PE classes. Additionally, there was a notable sex discrepancy observed, with 28% of boys participating in PE classes compared with only 18% of girls.

### Family and Peers

This indicator relied on data showing the influence of family members and peers in encouraging or limiting PA opportunities and participation among children and adolescents. Based on parental PA levels in the UAE, the Family and Peers indicator was assigned C− (62%) in 2016 and D− (20%‐26%) in 2022. In the 2016 Qatar Report Card, it was graded D based on the overall prevalence of physical inactivity, suboptimal dietary habits, and a high rate of obesity among the population. Lebanon did not have any data to inform this indicator; hence, it was marked INC.

### Government

This indicator was evaluated based on any evidence of governmental support in promoting and providing PA opportunities for children and adolescents through policy, legislation, or regulation. All 3 countries relied mainly on unpublished data to grade this indicator. Qatar assigned its highest grade to the Government indicator in both 2016 (B) and 2018 (B+). Similarly, in the UAE, the indicator was graded B+ across all 3 Report Cards. However, Lebanon’s grades were C+ in 2018 and D in 2022.

## Discussion

### Summary of Main Findings

This study addressed an important research gap by offering the first regional comparative synthesis of PA indicators in 3 Arab Middle Eastern countries with shared cultural and climatic contexts. It provides insights into a critical public health concern based on 25 years of data (1998‐2022). The findings revealed that less than one-third (15%‐33%) of children and adolescents participated in the recommended daily amount of PA (ie, an average of 60 minutes of moderate- to vigorous-intensity PA). Moreover, a large proportion (45%‐74%) exhibited high levels of sedentary behavior (ie, ≥2 hours of recreational screen time per day). Qatar and the UAE had comparable trends due to their similar demographics, geographic location, climate, and cultural characteristics. Compared with global estimates, PA levels in the 3 Arab Middle Eastern countries were close to those observed across other Asian countries participating in the AHKGA; however, they were generally lower than PA levels in other regions of the world.

### Interpretations, Implications, and Comparisons With Existing Literature

#### Human Development Index

Countries worldwide are classified by their level of development using the HDI, which measures a country’s success in 3 key dimensions: life expectancy, education levels, and the standard of living as measured by income per capita [30]. Global analysis of PA levels by HDI showed that countries with high and very high HDI generally had lower grades in behavioral indicators (ie, Overall Physical Activity, Organized Sports, Active Play, Active Transportation, and Sedentary Behavior) but higher grades in sources of influence indicators (ie, School, Family and Peers, Community and Environment, and Government) [36]. This pattern of lower PA and higher HDI was evident in most indicators (except for the Government indicator) across the 3 Middle Eastern countries included in our study.

On a global scale, the overall PA grades for countries with very high (≥0.800) HDI approximated a C, with the behavioral indicators near D and the source of influence indicators around B [33]. The UAE and Qatar grades were not far from the global averages of PA among other very high HDI countries participating in the Global Matrix initiative. Similarly, Lebanon’s grades were consistent with other participating countries classified as having a high HDI (eg, Thailand, China, Bulgaria, and several nations in South America), with most results predominantly around a grade of D [34].

#### Behavioral Indicators

The latest global insights from the Global Matrix 4.0 (2022) revealed an Overall Physical Activity average of D (27%‐33%) based on data gathered from 57 countries [13]. A study evaluating further temporal trends of Asian countries from Global Matrices 2.0‐4.0 showed consistently lower grades for Overall Physical Activity, with an average grade of D− (20%‐26%) across the years [20]. Countries in the Middle East have had similar and sometimes lower results (F to D) than regional (Asian) averages; however, the grades vary across the specific indicators.

The assigned grades for Sedentary Behavior across the 3 participating Arab Middle Eastern countries ranged between D− and C−, which is in line with the global findings observed over the past decade [37]. This was expected given the advancement in technologies and the widespread use of digital media. Moreover, the Organized Sport and Physical Activity indicator was marked INC in nearly all Asian countries (including Lebanon and the UAE) in Global Matrix 4.0 [13]. Considering its role in the overall health and PA of children and adolescents, it is important to incorporate organized activities into PA questionnaires with further specification of the type, intensity, frequency, and duration of each activity.

Active Play, known for its numerous health benefits in children and adolescents, remains one of the least researched and documented areas of PA [38]. This indicator is a critical component of children’s and adolescents’ physical, social, and cognitive well-being; however, it often goes unrecorded due to its unstructured nature [39]. In the most recent global reports (Global Matrix 4.0, 2022), Active Play was the indicator with the most INC grades (27/57, 47% INC; 30/57, 53% graded) [13]. Moreover, the average grades differed across countries participating in the Global Matrix in relation to the HDI; low-medium HDI countries scored around a C+, while high HDI countries scored around a D− [13]. This highlights the impact of the modern world regarding replacing unstructured PA with sedentary behavior. Country-specific barriers and enablers to assessing this indicator are worth exploring to better understand children’s total PA and design interventions that promote healthy, playful, and active lifestyles.

Low levels of Active Transportation in the 3 Middle Eastern countries may be partially explained by the transport infrastructure and urban design. None of the 3 countries have sufficient walking or cycling lanes dedicated to traveling long distances between residential communities and schools. Most cycling and pedestrian tracks are primarily designed for recreational use, rather than serving as active transportation routes. The region’s hot climate also adds to the barriers of outdoor PA in general, especially in Qatar and the UAE where temperatures can peak at 40.0 °C to 45.0 °C [32]. In Lebanon, the weather throughout the academic year is generally moderate (mean temperature 16.0 °C) [32], facilitating better conditions for active transportation. Additionally, the noticeable difference in Active Transportation between boys and girls may be due to cultural factors, such as the acceptability of female adolescents walking and cycling to and from school or other places. A study conducted in the Middle East and North Africa region identified a number of barriers and facilitators to PA among different subgroups of the population [40]. The most common barriers among children and adolescents were being female, older age, lack of time, and obesity. Other less common barriers included high screen time, inadequate school PE programs, lack of sports facilities, peer support, sleep, and dietary habits [40].

These findings highlight the relationship between different behavioral indicators and their joint effect on achieving optimal daily PA. Therefore, in order to improve PA levels among children and adolescents (girls and boys), the first step would be to develop and implement a systematic tool for the assessment of PA across different domains [41-43].

#### Physical Fitness Indicator

The dearth of data on physical fitness levels presents a critical public health concern, as it is an indicator that offers important insights into the overall health of children and adolescents and reveals early risk factors for chronic diseases that can develop later in life [44,45]. Routine evaluation and monitoring of physical fitness can also serve as a baseline national indicator of the efficacy of PA policies, educational interventions, and environmental support for promoting active lifestyles among children and adolescents. Since its introduction in the Global Matrix 3.0 (2018), Physical Fitness has been reported as INC in over one-half of the participating countries. Most grades for this indicator were assigned to countries classified as having high or very high HDIs, with a global average of C− [12,13].

Within the Middle East, Qatar has exhibited preliminary progress, where the Ministry of Education and Higher Education initiated a “Physical Fitness Evaluation for Public School Students” and published their report for the 2019‐2020 academic year online [46]. The results included 104,191 students (50% Qatari nationals) who were evaluated for cardiorespiratory fitness, strength endurance, flexibility, speed, and consistency. Based on these findings, normative values were established for each fitness component. Qatar did not participate in the latest Global Matrix; therefore, these data were not included previous Report Cards and will likely be included in future ones.

#### Sources of Influence Indicators

Globally, countries with very high HDIs typically scored the highest in the School indicator [36]. The most common grades assigned to this indicator among the 3 Arab Middle Eastern countries were D− and D, which are lower than the Asian and global averages (C+). It is worth noting that the School grades were partially affected by the variations in benchmarks used for grading throughout the Global Matrix iterations. Hence, it is difficult to directly compare this indicator across countries, regionally and globally. Having said that, many countries in Asia are still not performing well with school-related PA [19,20]. Although PE is mandated in most school curricula, the duration or frequency of classes does not always provide adequate amounts of PA to meet international guidelines. Additionally, the heavy focus on academic grades and achievements often causes students to neglect PE and overlook the importance of sports [20]. Given the benefits of PA to the overall well-being and academic performance of children and adolescents, it is essential for schools to place greater importance on PE classes and other sports within their curricula.

The influence of Families and Peers is multidimensional and demands extensive research for better understanding and assessment across regions and cultures [47]. Globally, this indicator had great variations (from INC to F to A+), with Asian countries globally scoring an average of C− [13,20]. Despite the recognized importance of social influences, the available data on this indicator remain limited and mostly incomplete in the 3 Arab Middle Eastern countries, mainly due to a lack of valid assessment tools. Community and Environment is another indicator that strongly influences children’s and adolescents’ engagement in PA and is assessed based on various factors such as local policies, infrastructure, the perception of safety, and access to green and blue spaces. Globally, the grades for this indicator varied between very high HDI countries (B) versus low, medium, and high HDI countries (C−) [36]. Further variations were observed across different regions such as Africa (D+), Asia-Pacific (C), Europe (B−), and the Anglosphere (B−) [13]. In Lebanon, Qatar, and the UAE, this indicator was marked INC across all Report Cards. This is due to a lack of consistent monitoring in these countries, which highlights a critical gap in understanding how the built and social environments support or hinder active lifestyles. To standardize the evaluation of these indicators, it is necessary to conduct periodic public household surveys aimed at assessing social and environmental factors related to PA. Addressing this gap is essential for informing urban planning and public health interventions and policies [48].

Finally, the government plays an important role in facilitating PA opportunities. In the UAE, the data suggested strong governmental support and leadership in promoting PA through national strategies, policies, and local initiatives. The “National Sports Strategy 2031” [49] aims to increase sports participation to 71% of the UAE population by enhancing access to both community-level and competitive sports. Further evidence included the “National Policy for Promoting Healthy Lifestyles” [50], “National Strategy for Wellbeing 2031” [51], and “Masar Project” [52]. Likewise, Qatar’s highest grades were assigned to the Government indicator in both Report Cards, reflecting the country’s commitment to supporting PA in the population. Some of Qatar’s strategies that prioritized PA for children and adolescents included the “Qatar National Sports Day” [53], “Qatar National Physical Activity Guidelines” [54], and “National Health Strategy 2018-2022” [55]. Additionally, the WHO-Qatar partnership (2021‐2024) on the FIFA (Fédération Internationale de Football Association) World Cup and health legacy played a significant role in advancing PA through global awareness campaigns [56]. Lebanon’s results for the Government indicator reflected moderate efforts from both governmental and nongovernmental stakeholders, based on data from the 2018 Report Card [26]. Although existing policies focus on enhancing PA in schools, the implementation and coverage remained limited, and there was no indication of sustainable funding, evaluation, or national legislation mandating these efforts. As a result, Government grades declined in the latest Report Card (2022) for Lebanon. To maximize the benefits of these governmental initiatives and programs, it is essential for all 3 countries to prioritize public education on the importance of PA across different domains.

### Report Card Team Recommendations for Improvement of Physical Activity Levels in the Region

In Global Matrix 3.0 (2018), Report Card members provided their top 3 priorities to improve the 10 common PA indicators in their respective countries. Based on these, we developed recommendations that are relevant to all 3 countries and the broader Middle East region for improving PA levels among children and adolescents.

#### Overall Physical Activity

We recommend developing and implementing PA surveillance programs using nationally representative samples, with the aim of measuring subjective and objective estimates of PA across different domains.

#### Organized Sport and Physical Activity

Our recommendations include improving access to affordable, well-structured organized sports within schools and communities; establishing talent identification programs and athlete development pathways that increase mass participation at the grass-roots level; and promoting PA participation among girls and children and adolescents with disabilities or chronic conditions.

#### Active Play

For this PA domain, we recommend the integration of active play into urban planning by creating safe, accessible, and culturally appropriate play environments and the development of relevant surveillance tools.

#### Active Transportation

Accelerating the development of safe and accessible infrastructures (eg, walking, running, and cycling path networks) within communities and cities could promote active transportation.

#### Physical Fitness

We recommend developing and implementing nationwide standardized physical fitness tests and monitoring programs in school curricula, paired with targeted interventions to improve fitness and physical performance levels.

#### Sedentary Behavior

Integration of regular active breaks in school lessons can interrupt long periods of sedentary behavior, and implementation of awareness campaigns could promote reductions in nonessential screen time.

#### School

We recommend the development of physical literacy among students and maximizing the time spent in MVPA (minimum of 150 minutes per week during school time across all schools and grades) while offering fun, enjoyable, and skill-developing activities for both boys and girls.

#### Family and Peers

Establishment of family-centered community PA initiatives and peer-led activity clubs in collaboration with municipalities and sports clubs are recommendations in this area.

#### Community and Environment

Our recommendations include continued development and maintenance of accessible green and blue active spaces (ie, parks and beaches) for all children and adults to interact socially as well as the development and implementation of related surveillance systems.

#### Government

Establishment of national multisectoral PA monitoring and accountability frameworks could support the implementation of health policies, PA guidelines, and school-based PA programs.

### Strengths and Limitations

To the best of our knowledge, this is the first study to synthesize and compare trends in PA indicators among children and adolescents in all Arab Middle Eastern countries participating in the AHKGA. The results were further compared with regional and global trends, offering valuable insights for policymakers. The study also highlighted major research and surveillance gaps and provided recommendations for the various PA indicators. Despite PA being an emerging area of research within the region, teams of PA researchers in each country retrieved and synthesized multiple data sources to grade several PA indicators over a 25-year period. When evaluating these grades and comparing between countries, we identified differences in data sources (ie, PA assessment was not harmonized across and within countries). This was carefully considered when comparing results and drawing conclusions.

The main limitation of this study was the lack of high-quality data for the majority of PA indicators (7/10, 70%) and the complete absence of data sources for the remaining 3 indicators (ie, Active Play, Physical Fitness, and Community and Environment). Specifically, there was a dearth of studies collecting accelerometer-derived objective estimates of PA using nationally representative samples. Certain indicators (eg, School, Family and Peers) were difficult to compare across the countries due to variations in the benchmarks used for grading. These challenges further point to the gaps in PA research within the region and the critical need for standardized and comprehensive PA surveillance studies.

### Conclusions

Children and adolescents in Lebanon, Qatar, and the UAE had low PA levels overall coupled with high levels of sedentary behavior. These results were consistent with regional findings and global trends. Overall, there was a dearth of data from representative samples in all 3 countries. There were significant gaps in specific indicators such as Active Play, Physical Fitness, and Community and Environment. Addressing the current challenges requires a thoughtful approach that considers the unique context of each country and promotes equitable access to opportunities for active living across the lifespan. There is a clear need for a standardized and comprehensive PA surveillance strategy that assesses both self-report and objective estimates of PA among children and their parents. School remains an important opportunistic arena for increasing PA levels. Enhancing the quantity and quality of PE programs and extracurricular sports would maximize the proportion of children and adolescents receiving the health benefits of regular PA. Stakeholder collaboration is essential for developing evidence-informed, locally relevant plans that address the current implementation gap between policy and practice. Specifically, this requires educational authorities, municipalities, environmental agencies, and sports councils to implement a harmonized approach to providing PA opportunities for all children and adolescents.

## Abbreviations

AHKGA: Active Healthy Kids Global Alliance
FIFA: Fédération Internationale de Football Association
GDP: gross domestic product
GM: Global Matrix
GSHS: Global School-based Health Survey
HDI: Human Development Index
INC: incomplete
MVPA: moderate- to vigorous-intensity physical activity
PA: physical activity
PE: physical education
UAE: United Arab Emirates
WHO: World Health Organization
